# In defence of the drug, two hearts, one profession: Reintegrating medicines expertise and patient-centred care in pharmacy's professional identity

**DOI:** 10.1016/j.rcsop.2026.100830

**Published:** 2026-08-17

**Authors:** Neal M. Davies

**Affiliations:** Faculty of Pharmacy and Pharmaceutical Sciences, University of Alberta, Edmonton, Alberta T6M 0L2, Canada

**Keywords:** Pharmaceutical care, Professional identity, Pharmacy education, Medicines expertise, Patient-centred care, Pharmaceutical sciences

## Abstract

For more than three decades, pharmacy has narrated its evolution as a movement away from the product and toward the patient. The pharmaceutical care era ushered in by Hepler and Strand in 1990 framed dispensing and product expertise as a chrysalis the profession needed to shed in order to mature. That framing has been useful, but it has also been misread, and the misreading has now been institutionalised. Some accreditation councils, regulatory and licensing bodies, and advocacy bodies have positioned the profession as one moving away from a so-called “product centric” past. The thesis of this commentary is otherwise. Pharmacy has never had a single heart; it has always had two, one beating for the drug and the other for the patient, and they have always beaten in the same chest, for four centuries and more. The pharmacist's claim to a place at the patient's bedside rests entirely on what the pharmacist knows about the molecule: medicinal chemistry, pharmaceutics, compounding, biopharmaceutics, pharmacokinetics, pharmacogenomics, radiopharmacy, pharmacognosy and now pharmaceutical artificial intelligence. The argument here is not nostalgic but expansive. Embrace them all. Without the drug, the diagnosis is a verdict, not a treatment; and without the pharmacist, no drug appears.

## 1 Two hearts

“Two hearts beat in pharmacy, and whoever silences one silences both.”

This commentary argues that pharmacy's professional identity understood here as the shared body of knowledge, values and practice boundaries by which a profession defines itself to its members, to other health professions and to the public has been narrowed by a persistent framing that treats medicines expertise and patient centred care as competing rather than complementary. Patient centred care, as used throughout this piece, means care organised around the needs, values and outcomes of the individual patient, whether or not that care involves the physical dispensing of a medication. The distinction matters beyond semantics: how a profession describes itself shapes what it teaches, what it is licensed to do, and what patients and other health professions expect of it. Alberta is used throughout as a case study, not because its experience is unique, but because its recent regulatory and advocacy documents illustrate, with unusual clarity, a pattern of framing that appears in varying degrees across other Canadian and international jurisdictions. This is a conceptual and argumentative commentary rather than a systematic review; documents and sources were selected to illustrate the pattern under discussion, not to comprehensively survey the field, and full citation details are provided throughout so that readers can assess the primary sources directly.

The dominant narrative since 1990 has been one of opposition: pharmacy has shifted, or must shift, from a “product focused” practice to a “patient focused” one.^1^, 2. Hepler and Strand themselves argued that pharmacy needed to mature beyond the apothecary role and accept responsibility for drug-therapy outcomes,^1^ a position rapidly endorsed by the American Society of Hospital Pharmacists, the American Association of Colleges of Pharmacy and the American Pharmacists Association.^2^ The intent of that vision remains sound. The misinterpretation came later, when subsequent generations of educators, regulators, advocacy organisations and policy makers began treating product expertise and patient care as if they sat on opposite ends of a single axis, where movement toward one necessarily means movement away from the other.

That framing is mistaken on its face. Hepler and Strand wrote that it is not enough to dispense the correct drug or to provide sophisticated pharmaceutical services^1^; they did not write that dispensing the correct drug had become unnecessary. The pharmaceutical care movement asked the profession to expand its object of accountability beyond the product, not to abandon the product itself; the care process, the object of professional attention and the dimensions of quality it recognised all widened together. Two hearts beat in pharmacy, to use the framing this commentary advances throughout. One pumps the molecule, the other pumps the patient relationship, and the same circulation feeds both. A profession that silences one heart will not survive on the other.

Pharmacists are not the only members of the animal kingdom that thrive on more than one heart: the octopus, the hagfish and the earthworm all sustain circulation through multiple pumps, a design that evolutionary biology treats as redundancy, not vestige.^3^, ^4^ Pharmacy's two hearts serve the same function; they are the design feature that has kept the profession circulating for four hundred years.

This commentary makes three claims. First, the dichotomy of “product centred” versus “patient centred” practice is false; the two are inseparable, and the international consensus, as articulated by the International Pharmaceutical Federation (FIP), the Accreditation Council for Pharmacy Education (ACPE) in the United States and major pharmaceutical thought leaders such as DiPiro, treats them so. Second, some recent articulation of pharmacy's future risks erasing the molecular substrate that gives pharmacy its identity, and does so in language that diverges from the international position. Third, the answer is not to retreat into a pre 1990 conception of dispensing, but to expand the territory of the molecule to include every domain that now defines it: medicinal chemistry, pharmaceutics, compounding, biopharmaceutics, pharmacokinetics, pharmacogenomics, radiopharmacy, pharmacognosy and pharmaceutical artificial intelligence. Each of these domains is the territory in which patient centred care is actually delivered.

## 2 Four centuries of two hearts

It is sometimes assumed that the patient centred era is a recent development in a profession that, before 1990, knew only the molecule. The historical record is otherwise. The Society of Apothecaries was incorporated by royal charter in 1617, separating apothecaries from grocers and giving them a distinct duty of care to the sick.^5^ The Edinburgh Pharmacopoeia of 1699 standardised preparations precisely so that patients across the realm could receive comparable medicines.^6^ The Apothecaries Act of 1815 in the United Kingdom required apothecaries to attend, prescribe for and supply patients, in effect codifying a primary care role two centuries before the term was coined.^5^ The very point of these guilded institutions was the patient. The molecule was the means; the patient was always the end.

Pharmacopoeial culture is older still. The Tang dynasty Xinxiu bencao of 659 CE, the Newly Revised Canon of Materia Medica, was the first state-sponsored pharmacopoeia and remained authoritative across East Asia for centuries.^7^ Avicenna's Canon of Medicine (1025 CE) and the Liber servitoris of Bulchasim Ben Aberazerim integrated drug preparation with patient indication.^7^ Long before the language of “patient centredness”, pharmacy was both. To frame the present generation as the first to bring the patient into pharmacy is to mistake renaming for invention.

What changed in 1990 was not the introduction of patient care into pharmacy; it was the re-articulation of the pharmacist's accountability for medication-related decisions and outcomes generally, not only for products that had physically left the dispensary. That re-articulation did not itself change the profession's existing identity; rather, it opened a door that some pharmacists and institutions then stepped through, expanding how that identity was exercised without replacing what it was built on. The two hearts have always been beating. What Hepler and Strand changed was the audibility of one of them.

## 3 The nine domains of the molecule

A pharmacist's claim to a unique role in healthcare rests on integrated mastery across nine domains, each of which is simultaneously a product science and a patient science. None of them can be amputated without consequence for patients. Each domain is examined briefly below.

### 3.1 Medicinal chemistry

Medicinal chemistry teaches the pharmacist why a molecule does what it does. Structure-activity relationships, prodrug activation, chiral selectivity, and physicochemical determinants of permeability and solubility are not abstractions; they are the basis on which a pharmacist can advise that ramipril and lisinopril are not interchangeable in a patient with hepatic dysfunction, or that fexofenadine, the carboxylic acid metabolite of terfenadine, was a safer second generation antihistamine because the parent had been responsible for QT prolongation through hERG-channel blockade.8., 9 A pharmacist without a working knowledge of medicinal chemistry lacks the language in which prescriber queries are usefully answered.

### 3.2 Pharmaceutics

Pharmaceutics is the discipline of formulation and delivery, and it is the discipline most directly responsible for the difference between a drug and a medicine. The lipid nanoparticle technology that delivered the COVID-19 mRNA vaccines was, at the bedside, an intervention as critical as any clinical decision made about a patient.^10^ Self-emulsifying drug delivery systems, transdermal patches, inhaled biologics, controlled release matrices and depot injections are all instances of pharmaceutics meeting the patient. A pharmacist who does not understand why a sustained-release dosage form should not be crushed cannot safely manage a patient receiving that dosage form through an enteral feeding tube.

### 3.3 Compounding

Compounding is the oldest pharmaceutical activity, and despite the rise of industrial manufacturing it remains essential. A neonate weighing 800 g cannot receive a tablet. A child with a swallowing disorder requires a suspension. A patient with multiple drug allergies may require a preservative-free preparation. Patients in long term care receive extemporaneously compounded eye drops, mouthwashes, suppositories and topical preparations daily.11., 12 The National Association of Pharmacy Regulatory Authorities (NAPRA) in Canada has, in its Model Compounding Competencies and its 2024 Entry to Practice Competencies, required that every pharmacist demonstrate proficiency across non sterile, sterile and hazardous compounding.13., 14. The regulator has not abandoned the product. Neither should the educator.

### 3.4 Biopharmaceutics and pharmacokinetics

Biopharmaceutics, including the Biopharmaceutics Classification System and biorelevant dissolution science, and clinical pharmacokinetics, including population modelling, model informed precision dosing and therapeutic drug monitoring, are the disciplines that translate dose into exposure and exposure into effect.^15^, ^16^ Vancomycin AUC guided dosing, busulfan target attainment in stem cell transplantation, voriconazole trough monitoring and tacrolimus area-under-the-curve targeting are all routine pharmacist activities that are unintelligible without quantitative pharmacokinetics.^16^, ^17^ These competencies are explicitly named in NAPRA's entry-to-practice competencies and in ACPE's 2025 Standards.13., 18

### 3.5 Pharmacogenomics

Pharmacogenomics has moved from research curiosity to standard of care for drugs including abacavir (HLA-B*57:01), carbamazepine (HLA-B*15:02 in Asian populations), thiopurines (TPMT and NUDT15), clopidogrel (CYP2C19) and several antidepressants (CYP2D6 and CYP2C19).^19^, ^20^ The Clinical Pharmacogenetics Implementation Consortium has issued more than thirty guidelines that translate genotype to phenotype to dose, with the 2022 update on CYP2C19 and clopidogrel exemplifying how pharmacogenomic data flow into individualised antiplatelet selection.^20^, ^21^ Pharmacogenomics is patient centred prescribing at the molecular level.

### 3.6 Radiopharmacy and theranostics

Radiopharmacy and theranostics, the dual diagnostic and therapeutic use of radiolabelled molecules, have entered routine oncology with [^177^Lu]Lu-DOTATATE for somatostatin-receptor-positive neuroendocrine tumours (NETTER-1), [^177^Lu]Lu-PSMA-617 for metastatic castration-resistant prostate cancer (VISION), [^223^Ra]radium dichloride for prostate cancer bone metastases and [^131^I]MIBG for refractory neuroblastoma.^22^, ^23^ Each requires a pharmacist who understands radioactive decay, radiation safety, kit preparation, dosimetry and patient counselling on radiation precautions. The Lancet Oncology Commission on Radiotherapy and Theranostics and its companion review on workforce challenges have documented that the global pharmacy workforce is unprepared for the rapid clinical expansion of theranostics, and that Canada in particular is losing its nuclear pharmacy expertise to retirement without succession.^24^, ^25^ Radiopharmacy is patient centred care for the patient with cancer; it is not optional.

### 3.7 Pharmacognosy and natural products

Pharmacognosy, the science of drugs from natural sources, has been quietly removed or compressed in many North American pharmacy curricula since the 1980s, on the implicit assumption that synthetic chemistry had retired it.^26^ That assumption has aged poorly. Roughly a quarter of approved small molecule drugs in any given decade are natural products or natural-product derivatives, including paclitaxel from *Taxus brevifolia*, the statin family from *Aspergillus* species, artemisinin from *Artemisia annua* (the discovery for which Tu Youyou, a pharmacist, was awarded the Nobel Prize in 2015).^27^ The case for restoring pharmacognosy to the entry to practice curriculum is now overwhelming: the cannabinoid pipeline requires graduates literate in plant chemovar and terpene chemistry, and the renaissance of psilocybin, MDMA, ketamine, kratom and ibogaine for psychiatric indications requires graduates who understand alkaloid chemistry. The botanical drug regulatory pathway codified by the United States Food and Drug Administration's Botanical Drug Development Guidance for Industry (revised 2016) presumes a workforce literate in pharmacognosy.^28^ If the historical name has fallen out of fashion, the discipline should return under whichever banner the curriculum can carry; natural products science, natural health products, plant-derived therapeutics. The science is the same and the importance is undeniable. Heinrich, writing in the American Journal of Pharmaceutical Education in 2012, called pharmacognosy a discipline that has not gone away even as its institutional position has eroded.^26^ That call is, if anything, more urgent now than then.

### 3.8 Pharmaceutical artificial intelligence

Pharmaceutical artificial intelligence has arrived rapidly and unevenly. Large language models support clinical decision support and drug information triage; machine learning models support ADMET prediction, formulation optimisation and population pharmacokinetic estimation; reinforcement learning approaches support adaptive dosing; transformer architectures generate candidate scaffolds for medicinal-chemistry exploration.^29^, ^30^ The American Society of Health-System Pharmacists has issued a 2024 statement on the use of artificial intelligence in pharmacy.^31^ The pharmacist who is to use these tools safely must understand both the clinical question and the molecular logic the model is approximating. A pharmacist deprived of the molecular sciences cannot evaluate a model's output critically. Pharmaceutical AI does not relieve pharmacy of the pharmaceutical sciences; it raises the demand for them. The pharmacist's role in pharmaceutical AI is not to be replaced by it but to govern it. The drug that computes still requires a human who understands the drug.

### 3.9 Pharmacology and toxicology: the central nervous system of the octopus

Pharmacology and toxicology bind the other domains together. Receptor occupancy, signal transduction, dose response, mechanism based toxicity and idiosyncratic adverse reactions are the language in which the patient encounter is, in fact, conducted. Without pharmacology, every other domain becomes either a craft or a catalogue. Pharmacology and toxicology are not a ninth arm of the octopus; they are its central nervous system. The eight arms above coordinate themselves through pharmacological reasoning. Sever this nervous system and the arms become uncoordinated; the integration on which the pharmacist's identity rests collapses.

“Whoever surrenders the molecule surrenders the patient.”

## 4 Drug expertise is patient care

The strongest argument against the “patient versus product” framing is that the most consequential patient centred decisions in modern healthcare are themselves drug decisions. Pharmacist led medication review, deprescribing, antimicrobial stewardship, anticoagulation management, oncology supportive care, individualised dosing and pharmacogenomic counselling are not soft, communicative activities that exist alongside drug knowledge; they are drug knowledge applied at the bedside.^32^, ^33^ A pharmacist who reviews a polypharmacy regimen without a working understanding of CYP450 induction, protein binding displacement, renal clearance and active metabolite accumulation is not practising patient centred care; that pharmacist is practising checklist centred care, and the patient is the worse for it.

Preventable adverse drug events remain a leading cause of avoidable hospital admission and death.^32^, ^33^ Systematic review evidence shows that pharmacist led interventions in primary care reduce hospital admissions related to medication related adverse events,^33^ and large meta analyses of pharmacist participation in vancomycin therapeutic drug monitoring show reduced acute kidney injury, improved target attainment and lower mortality.^34^ The mechanism by which pharmacists generate that benefit is, at root, their command of pharmaceutics and pharmacokinetics, and increasingly of pharmacogenomics and computational decision support. When that command is hollowed out, the intervention loses its statistical signal. Drug expertise is not a complement to patient care; it is the engine of it.

## 5 Cases the pharmacist could not hand off

“The argument is settled at the bedside, not in the class room or boardroom.”

Abstract argument is necessary but insufficient. Three cases follow, each anchored in peer reviewed literature, each chosen because in each case the patient's outcome turned on a body of knowledge that no other healthcare professional possesses in the same combination. None of these cases is hypothetical. None could have been resolved by a competent diagnosis alone, by a good bedside manner alone, or by an algorithm alone. Each was, in the end, the pharmacist's case.

Case 1 The crushed extended release tablet.

Schier and colleagues, writing in the Annals of Pharmacotherapy in 2003, reported the death of a 38 year old woman after a crushed extended release nifedipine tablet was administered through her nasogastric tube together with labetalol. Crushing the controlled release matrix converted a 24 h vasodilator dose into an immediate dump of free drug; in combination with the beta-blocker, it produced fatal hypotension and asystolic arrest.^35^ Cornish's CMAJ “Avoid the crush” commentary confirms this is a common, preventable hazard typically intercepted by pharmacists.^36^ The pharmacist's reasoning here is pharmaceutics: the dosage form *is* the dose, and altering the dosage form alters the pharmacokinetics in ways that no diagnosis only clinician will reliably predict. Without the pharmacist, the patient receives a fatally rapid release of a drug whose label promised twenty four hours of safety.

Case 2 Vancomycin in the septic patient with borderline renal function.

An adult patient with methicillin resistant *Staphylococcus aureus* bacteraemia is started on empirical vancomycin. Trough only monitoring at 15 to 20 mg/L was the historical norm; the 2020 ASHP, IDSA, PIDS and SIDP consensus guideline replaced trough only monitoring with AUC guided dosing in the range 400 to 600 mg·h/L, on the basis that aggressive trough targeting was associated with avoidable nephrotoxicity.^17^ Meta-analyses confirm that pharmacist involvement in vancomycin therapeutic drug monitoring is associated with reduced acute kidney injury, improved target attainment and reduced mortality.^34^ The pharmacist's reasoning here is clinical pharmacokinetics, deployed quantitatively: estimating the AUC from a peak and trough or from a Bayesian forecasting model, adjusting for changing creatinine clearance and integrating the result with susceptibility data. No other profession is taught this calculation as a core competency. Without the pharmacist, the patient receives more nephrotoxicity, more treatment failure and more avoidable death.

Case 3 The pediatric patient with no commercial formulation.

A two year old with a rare metabolic disorder requires a medication that is available commercially only as an adult strength tablet. Crushing the tablet would deliver an unpredictable and potentially toxic dose; splitting it would be inaccurate; flavouring it for pediatric acceptance would be impossible without an excipient compatibility assessment. The compounding pharmacist prepares an oral suspension at the correct strength, with documented stability, with an excipient profile free of the patient's known allergens and at a flavour the child will accept.11., 12 Carvalho and Almeida's review confirms such customisation improves adherence and outcomes in populations otherwise excluded from rational pharmacotherapy, an unmet need only compounding can address.^37^, ^38^ The pharmacist's reasoning here is pharmaceutics, biopharmaceutics and stability chemistry combined. Without the pharmacist, the child is either undertreated, mistreated or untreated. There is no other professional in the system who can compound the suspension, and no algorithm that can replace the bench.

## 6 Without the pharmacist, no medication

“The diagnosis without the drug is a verdict, not a treatment.”

Around the patient there is a kind of choreography. The physician examines, names and explains. The nurse practitioner inspects, listens and reassures. The clinical pharmacist, too, can join that dance, and increasingly does, with the addition of a diagnosis through expanded scopes of practice in many jurisdictions.2., 39 But the patient does not heal from the dance. The patient heals from the medication, and *without a pharmacist no medication appears*. No prescription, however brilliantly arrived at, treats the patient until it is dispensed, formulated, reconstituted, irradiated or compounded; until it is checked for stability and incompatibility; until its dose is interpreted in the light of the patient's renal function, hepatic function, age, weight, genome and concurrent therapy. Every act of patient care that uses a medicine, and almost all do, depends on a pharmacist somewhere in the chain. Erasing that chain in pursuit of a patient only narrative would not produce more care; it would produce no care.

## 7 A brief portrait of the consensus

A particular institutional consensus appears to be forming that frames pharmacy's evolution as a movement away from the product. The Canadian Council for Accreditation of Pharmacy Programs (CCAPP)’s Educational Outcomes for the First Professional Degree describe a graduate prepared to deliver patient focused care.^40^ The Alberta College of Pharmacy (ACP)’s Standards of Practice for Pharmacists and Pharmacy Technicians, in commentary accompanying the standards, describe person centred care as care delivered independent of, and in some formulations explicitly not through, a particular medication or product transaction.^41^, 42. In ACP's own words, explaining the standards to members: “We wanted to make the standards person-centred and not prescription-centred.” The Joint Alberta Pharmacy Vision, signed by ACP, RxA, the Pharmacy Technician Society of Alberta and the Canadian Society of Hospital Pharmacists Alberta Branch, refers to a 10 year roadmap for moving away from a “product centric” model.^42^ RxA's public advocacy materials describe the pharmacist's value as resting in expanded scope, rather than in molecular expertise.^44^

The substance of these documents is, in many places, admirable. The aspiration to recognise pharmacists as primary care providers, to expand prescribing authority, to formalise ambulatory ailments management and to compensate pharmacists for cognitive services is not in dispute here. What is in dispute is the rhetorical frame: that pharmacy must move away from something it has been, in order to become something it should be. The remainder of this commentary argues that this frame is wrong, that it is not shared by the international consensus and that it will, if not corrected, narrow the very identity it claims to expand.

## 8 Reading the consensus: international to local

To read the “patient-centred” consensus accurately, it must be set against the wider field of voices that shape pharmacy at successive levels of authority. The discussion that follows therefore moves, in deliberate order, from the international position (FIP) to the accreditation bodies that govern pharmacy education in the United States and Canada (ACPE and CCAPP), then to the Canadian national professional voice, the Canadian Pharmacists Association (CPhA), then to the Canadian regulators, the national standard setter (NAPRA) and the provincial licensing authority in the author's jurisdiction (ACP), then to the provincial advocacy body (RxA) and finally to the universities themselves. At every level, the question is the same: does the body in question treat the product and the patient as alternatives, or as the same thing seen from two angles?

### 8.1 The international position: FIP

The International Pharmaceutical Federation, the global federation of national pharmacy organisations and the publisher of policy that shapes pharmacy practice across more than 150 countries, uses neither the language of “product centric” pharmacy nor the language of a profession that has shed the product. FIP's 2022 Statement of Policy on People-Centred Pharmaceutical Care begins from the Hepler and Strand definition and explicitly links it to the optimisation of medication therapy.^45^ The pharmacist, on FIP's account, takes responsibility for optimising medication therapy and improving patient health, the two phrases held together as a single sentence. People centred care.

FIP's Development Goals, published in 2020 and refined in 2024, make this division of labour explicit at the structural level. Development Goal 14 is Medicines Expertise. Development Goal 15 is People-Centred Care. They sit side by side, not in competition, and the FIP Global Competency Framework requires both at foundation level.^46^, 47. This is exactly the two-hearts position; the international body has codified it.

### 8.2 Accreditation standards: ACPE and CCAPP

The Accreditation Council for Pharmacy Education governs accreditation of Doctor of Pharmacy programs in the United States. Its 2025 Standards, effective 1 July 2025, retain the pharmaceutical sciences as a named curricular core. Standard 2 names medicinal chemistry, pharmaceutics, biopharmaceutics, pharmacokinetics, pharmacology, toxicology, pharmacogenomics and pharmaceutical compounding as required content domains, alongside therapeutics, social and administrative pharmacy and the patient care competencies.^18^, ^48^ ACPE has not chosen between product and patient. It has required both. The development paper for the 2025 Standards, by Kiersma and colleagues, makes the integration explicit; the standards are designed to produce graduates who deliver patient care through pharmaceutical science expertise, not in spite of it.^48^

CCAPP, the Canadian counterpart to ACPE, takes a different rhetorical approach in its current Educational Outcomes for the First Professional Degree.^40^ The pharmaceutical sciences are present, but they are present diffusely, distributed across competencies rather than named as a curricular core, and the framing of the document repeatedly emphasises practising, patient focused pharmacists as the desired graduate. The document does not name medicinal chemistry, pharmacognosy, radiopharmacy or pharmaceutical artificial intelligence as required content. The 2025 ACPE document does. The contrast is instructive. CCAPP's rhetorical trajectory has moved closer to that of the patient centred consensus than to that of the international and American positions, and it has done so without an explicit appendix of required pharmaceutical science content.

### 8.3 The Canadian national voice: CPhA

The Canadian Pharmacists Association is the national voice of pharmacy in Canada. Founded in 1907, it speaks for pharmacists in community, hospital, academic, government and industry settings, and it publishes the flagship Canadian drug information resources RxTx and the Compendium of Pharmaceuticals and Specialties.^49^ These two product information resources are CPhA's most used outputs, undercutting daily any notion that the national voice has abandoned the product.

CPhA's explicit vision, articulated in the Blueprint for Pharmacy and reaffirmed in successive policy documents, is optimal drug therapy outcomes for Canadians through patient centred care.^50^ The preposition is the entire argument; it is through, not instead of. CPhA's 2024 Transforming Primary Care in Canada white paper, by Liu, Paes, Al Hamarneh and Tsuyuki, similarly frames pharmacy as a first point of contact for primary care that leverages education, skills and expertise to deliver high-quality care.^51^ That education, skill and expertise rests substantially on the pharmaceutical sciences. CPhA has positioned them as the foundation of the primary care role, not as something to be left behind in the journey to it.

### 8.4 The Canadian regulators: NAPRA and ACP

NAPRA, the alliance of Canadian provincial and territorial pharmacy regulatory authorities, sets the model standards on which provincial licensing bodies such as the Alberta College of Pharmacy (ACP) draw. Together they make the integration of product and patient explicit at the regulatory level.

NAPRA's Professional Competencies for Pharmacists and Pharmacy Technicians at Entry to Practice in Canada (October 2024) names compounding, pharmacokinetic interpretation, drug-interaction assessment, formulation knowledge and pharmacogenomic application as required entry to practice abilities, sitting alongside patient assessment, communication and care planning competencies.^13^ Its Model Compounding Competencies (2022, republished 2024) formalise non sterile, sterile and hazardous compounding as a demonstrated competency for every pharmacist, not a retired one.^14^ And its Model Standards of Practice (2022 revision) state that the needs of the person seeking healthcare are the priority, met through medication appropriateness, supply, safety and follow-up: the patient at the centre, the molecule the means by which the centre is reached.^52^

ACP, the regulatory and licensing authority for pharmacists and pharmacy technicians in Alberta, describes person centredness as the heart of its Standards of Practice, effective 1 February 2025.^43^ The substance is unobjectionable, but some of the College's accompanying commentary is less settled: an ACP News piece characterised person-centred care as care delivered independent of the medication transaction, phrased as “and not” rather than “and.”^41^ That framing sits at variance with FIP's, CPhA's and NAPRA's integration, and signals a movement away from the product that none of the international, national, accreditation or standard-setting voices endorse. The corrective is rhetorical, not procedural: person-centred care is not a domain competing with the other seven; it is the orientation through which all seven are practised.

Alberta's regulated pharmacy technicians, licensed by ACP under the same Health Professions Act framework, hold a distinct and narrower scope focused on the technical and product related elements of practice; they do not independently assess patients, prescribe, adapt therapy or take clinical responsibility for care outcomes. The dual role this commentary defends, molecular expertise joined to direct patient care, remains specific to the pharmacist.

### 8.5 The provincial advocacy body: RxA

Provincial advocacy bodies, including the Alberta Pharmacists' Association (RxA), typically position the pharmacist's unique value in terms of an expanded scope of practice that overlaps with that of physicians and nurse practitioners.^44^ Scope expansion is a legitimate advocacy goal. It is not, however, an identity. Identity is what survives when scope is contested. If the pharmacist's value rests in scope alone, then any other professional with a comparable scope will be a substitute, and the political economy of healthcare will, in time, treat them as such.

The pharmacist's identity rests on integrated mastery of the molecule across all domains. Compounding, formulation, pharmacokinetics, pharmacogenomics, radiopharmacy, pharmacognosy and pharmaceutical artificial intelligence are not delegatable to a prescribing physician or a nurse practitioner. They are what the pharmacist alone does. Advocacy of this kind will be sturdier if it claims, on behalf of pharmacists, the entire pharmaceutical chain as the territory in which patient centred care is delivered.

### 8.6 Universities and curricular practice

Curricular practice in two American flagship programs illustrates how the integration is realised at the entry-to-practice level. The University of Houston College of Pharmacy organises its PharmD curriculum around integrated organ-system modules in which pathophysiology, pharmacology, medicinal chemistry and therapeutics are taught together.^53^ The University of Michigan College of Pharmacy has, since 2014, integrated medicinal chemistry and pharmacology into a single Principles of Drug Action course sequence that runs throughout the curriculum.^54^ These are accredited, well regarded programs, not anachronisms.

DiPiro and colleagues, in DiPiro's Pharmacotherapy: A Pathophysiologic Approach, now in its thirteenth edition, structure the entire textbook around the integration of pathophysiology, pharmacology, medicinal chemistry and therapeutics, with each disease chapter opening with a Pharmacists' Patient Care Process.^55^ DiPiro, speaking at the 2024 National Academies workshop on Innovations in Pharmacy Training and Sustainable Practice, argued that pharmacy must continue to adapt and evolve, and explicitly named exposure to interprofessional practice, technology-enabled care and clinical decision making as additions to, not replacements for, the pharmaceutical science core.^56^ AccessPharmacy, the McGraw-Hill platform that hosts DiPiro's work, organises its curricular hierarchy around medicinal chemistry, pharmaceutics, pharmacoepidemiology, pharmacogenomics, pharmacognosy, pharmacokinetics, pharmacology and pharmacotherapy as parallel sciences, all required, none subordinate.^57^ Beyond curricular structure, other thought leaders, including Zellmer, Knapp and Holdford, have made a related argument over the past two decades: that the pharmacist's value to society is rooted in distinctive knowledge of medicines, a resource worth sustaining for the patients who depend on it, not a territory to be defended for its own sake.^58^, ^59^

Canadian faculties have, in some cases, made parallel commitments: the University of Manitoba's PharmD curriculum, for example, builds its clinical therapeutics courses directly on top of its pharmaceutical sciences courses, with a dedicated pharmacokinetics-pharmacodynamics course integrated into patient-facing therapeutic decision making rather than taught as a separate, self-contained subject.^60^ The integration model is available, accredited, evidence based and proven; the only barrier to embracing every domain of the molecule is rhetorical.

## 9 Counterarguments and limitations

Four schools of thought deserve acknowledgement: three sympathetic, one openly antagonistic and answered here with greater force.

First, the curricular pendulum had to swing toward patient care because pre-1990 graduates too often could not interview a patient or communicate with a prescriber. This is a fair historical caution, but the pendulum metaphor is wrong: the two hearts beat together, and strengthening one by weakening the other is no fitter than the opposite error forty years ago.

Second, pharmacy curricula are already overloaded; something must give. This is true. The remedy is integration, not amputation: Houston and Michigan show medicinal chemistry, pharmacology and therapeutics can be co-taught in less time than parallel silos require. Compression into clinically anchored modules is a curricular improvement; excision is not.

Third, patient centred language may be defended as politically and economically necessary, since pharmacist remuneration often depends on demonstrating value beyond the dispensing transaction. The remedy remains rhetorical: remuneration argued from integrated molecular mastery is harder to substitute away than remuneration argued from having moved beyond the product.

Fourth, and the position to which the present commentary is most directly opposed, a vocal strand of the recent pharmacy education literature has begun to argue that pharmacy must purge the “product centric” identity altogether. Wagner and colleagues, writing in the *American Journal of Pharmaceutical Education*, propose that pharmacy resolve its identity crisis by “letting go of past identities”, with the BSPharm framed as the product centric past and the PharmD as the patient centric present.^61^ Al Diery and Hussain, in *Person centred care in pharmacy: time to walk the talk*, demand that pharmacists evolve from “passive dispensers to proactive engagers” and accuse the profession of being trapped in a risk averse culture that “positions pharmacists as gatekeepers of medication information” rather than as co designers of care.^62^ Nelson and colleagues, in the *Journal of the American College of Clinical Pharmacy*, attempt the kinder version of the same move by relocating the pharmacist's identity from the molecule to the abstract terrain of “medication therapy problems”, a frame in which the molecule itself is conspicuously demoted.^63^ Comparable rhetoric pervades the consultancy and trade press of the pharmaceutical industry, where “a departure from traditional approaches, which have often been more product or profit focused” is now sold as a strategic imperative.^64^

Each of these positions deserves a forceful answer, because each, in its own way, mistakes a costume change for an identity. The Wagner thesis treats the apothecary, the dispenser, the merchandiser and even the “expert advisor” as legacy roles that the modern pharmacist must outgrow. The conceit only works if one ignores what the patient at the bedside actually needs, which is, in nearly every case, a competently composed and verified medicine. Pharmacists are not embarrassed by the apothecary; the apothecary is the reason there is a pharmacist. The Al Diery and Hussain thesis presumes that the choice is between “passive dispensers” and “proactive engagers”, but a pharmacist who reformulates a pediatric suspension, calculates a vancomycin AUC, prepares a [177Lu]Lu PSMA 617 dose or interprets a CYP2C19 genotype is doing none of these things passively, and the framing slanders the very practice it claims to elevate. The Nelson thesis is more sophisticated and, for that reason, more dangerous; an identity defined by “medication therapy problems” rather than by the medicine itself can be ported, with very little resistance, to any prescriber willing to learn the language. An identity defined by integrated mastery of the molecule cannot.

There is a deeper error to name. The patient centric movement, in its more zealous formulations, treats the prescription as a moral hazard, as if to dispense a medicine were to fail at care and that it is beneath a pharmacist and left to a lowly pharmacy technician or assistant. This inverts the logic. The pharmacist's entire claim to a patient's trust rests on the patient receiving a medicine that is the right molecule, the right dose, the right route, the right formulation, the right schedule and the right counsel. Strip the pharmacist of the molecule and what remains is a generic communicator with a longer training program than is needed for the role. No profession secures its future by surrendering the only thing it does that no one else can do. *The Pharmaceutical Journal*'s elevation of “person centred over patient centred” care, sold as “not just semantics”, is in fact precisely semantics: a relabelling exercise that does not bring a single pharmacist nearer the bedside but does invite every other healthcare worker to claim the same vocabulary.^65^ The position of this commentary is the opposite. The pharmaceutical is not the past from which the profession must escape. The molecule is the only ground on which patient centred care can stand. To argue otherwise is to argue, in the end, against the profession itself.

## 10 The pharmacist's unique value, stated plainly

The pharmacist's unique contribution to healthcare can be stated in a single paragraph, and it is worth stating it that way. The pharmacist is the healthcare professional whose education, regulation and identity rest on integrated mastery of the molecule across nine domains: medicinal chemistry, pharmaceutics, compounding, biopharmaceutics and pharmacokinetics, pharmacogenomics, radiopharmacy, pharmacognosy and pharmaceutical artificial intelligence, all delivered to and for patients in person centred clinical encounters. No other professional shares that integration. The physician knows some pharmacology and therapeutics but not, as a rule, formulation, compounding, radiochemistry or biopharmaceutics. The nurse practitioner shares scope in prescribing but not the molecular substrate. The chemist knows the molecule but not the patient. The pharmacist is the integrator. This integration is the profession's economic moat, its political defence and its claim on a place at the bedside. Surrender it and the profession surrenders the case for its existence.

## 11 Considerations for stakeholders

The considerations below are offered for discussion, not as directives. They touch, in turn, on the international body, the accreditation councils, the national professional voice, the national association of regulatory authorities, the licensing authority, the advocacy body, the joint visioning partners and pharmacy education programmes. None is intended as criticism of a specific body; each reflects a broader pattern this commentary has traced across levels of pharmacy governance, and the body named in each case is simply the level at which that pattern is most visible. The argument is not against any of these bodies, but against a rhetorical drift that, left unaddressed, risks diminishing a profession in which all of them, whether through regulation, advocacy, accreditation or education, have a stake.

### 11.1 Consideration 1

There may be value in continuing to articulate Development Goal 14 (Medicines Expertise) and Development Goal 15 (People-Centred Care) as inseparable rather than alternative goals, and in the FIP Global Competency Framework naming pharmacognosy and pharmaceutical artificial intelligence explicitly as foundation-level competencies, on a par with medicinal chemistry, pharmaceutics and pharmacokinetics. Policy statements published in journals within FIP's family could continue to lead the international position that the product and the patient are not opposites.

### 11.2 Consideration 2

ACPE's explicit pharmaceutical science appendix, naming medicinal chemistry, pharmaceutics, biopharmaceutics, pharmacokinetics, pharmacology, toxicology, pharmacogenomics, pharmaceutical compounding, radiopharmacy, pharmacognosy and pharmaceutical artificial intelligence as required content, is worth maintaining; CCAPP restoring an equivalent appendix would bring the two standards closer into alignment. The Canadian “practising patient focused graduate” language might likewise be recast as a practising pharmacist who integrates medication expertise with person centred care.

### 11.3 Consideration 3

The “through, not instead of” formulation already present in CPhA's Blueprint for Pharmacy offers a useful anchor that successive policy documents could continue to build on, making explicit that pharmacy's value to Canadians is delivered through molecular mastery exercised at the bedside. RxTx and the Compendium of Pharmaceuticals and Specialties are visible artefacts of that mastery and could be articulated more directly as the foundation of the primary care role.

### 11.4 Consideration 4

NAPRA's entry to practice and compounding competencies already integrate product and patient; adding explicit pharmacogenomics, radiopharmacy, pharmacognosy and pharmaceutical artificial-intelligence competencies at the next revision would extend that integration to map cleanly onto the nine domains discussed above, and would give the provincial regulatory authorities a clearer national reference point to implement.

### 11.5 Consideration 5

At its next review, the Standards of Practice for Pharmacists and Pharmacy Technicians could describe person centred care as delivered through a competently composed prescription, rather than as independent of it; retiring the conjunction “and not” in favour of “through” would achieve much of this on its own. Person centred care is the orientation through which the other domains of practice are practised, not a domain defined in opposition to the prescription, which remains one of its most common vehicles.

### 11.6 Consideration 6

Advocacy framing that articulates the pharmacist's unique value in terms of the knowledge and skills the pharmacist holds that no other health professional duplicates, integrated mastery of the molecule across compounding, formulation, pharmacokinetics, pharmacogenomics, radiopharmacy, pharmacognosy and pharmaceutical artificial intelligence, may prove sturdier over time than framing centred on scope alone. Scope alone is not identity, though the two are related; identity is what tends to survive when scope is contested.

### 11.7 Consideration 7

The next iteration of the joint 10 year roadmap offers an opportunity to revisit the rhetoric of shifting away from a “product centric” model in favour of the language of integration: pharmacy as the profession in which product expertise and person centred care are inseparable, with the entire pharmaceutical chain claimed as the territory in which patient centred care occurs.

### 11.8 Consideration 8

Pharmacy education programmes have an opportunity to resist curricular amputation by integrating the pharmaceutical sciences with therapeutics through organ system or disease state modules, as Houston, Michigan and Manitoba have each done, so that medicinal chemistry, pharmacokinetics, pharmacogenomics, radiopharmacy, pharmacognosy and pharmaceutical artificial intelligence are taught as clinical sciences with bedside applications. Restoring pharmacognosy, repackaged as natural products science if the historical name is unfashionable, and embedded in the cannabinoid, psilocybin and botanical drug pipelines, would strengthen rather than weaken curricular rigour. Quantitative pharmacokinetic and biostatistical skill development is worth maintaining alongside this integration, not at its expense. A recent narrative review of curricular and accreditation imperatives for an artificial-intelligence-augmented pharmacy profession reinforces this recommendation.^66^

## 12 Conclusion

The pharmacist's claim to a place at the bedside rests on what the pharmacist knows about the molecule, joined to the social, behavioural and administrative competencies through which that knowledge reaches the patient. To frame this as a movement away from the molecule is to misunderstand the profession's history, to break with the international consensus and to surrender the case for the profession's continued existence. The recommendation of this commentary is not nostalgic. It is expansive. Embrace medicinal chemistry. Embrace pharmaceutics, compounding, biopharmaceutics and pharmacokinetics, pharmacogenomics, radiopharmacy, pharmacognosy, pharmacology and toxicology, and pharmaceutical artificial intelligence. Nine domains, eight arms and a nervous system, one octopus, two hearts. Embrace patient centred care, in which all of these are exercised. Embrace, in short, both hearts. Other animals have done it; the octopus, the hagfish and the earthworm survive precisely because they did. Pharmacy will too, if it remembers that it has, all along.

There is a final word to be said for the pharmaceutical sciences themselves. They are the source of every medicine the profession dispenses, every dose it adjusts, every adverse event it forestalls and every guideline it implements. They are the reason the pharmacist exists as a distinct profession at all. Their defence is not nostalgia; it is professional self-preservation in an era in which other healthcare professionals have begun to claim the patient care territory that pharmacy too readily surrendered as its own. Pharmacy, properly understood, is the profession of the molecule in the service of the patient. That has always been its definition, and it is the only definition on which its future can be built. The drug is not the past from which the profession must escape; it is the foundation on which everything the profession does for patients still rests.

## CRediT authorship contribution statement

**Neal M. Davies:** Writing – review & editing, Writing – original draft, Visualization, Validation, Resources, Methodology, Investigation, Formal analysis, Conceptualization.

## Declaration of competing interest

The author declares there are no competing interests or conflicts of interest.
